# Microarray analysis of potential biomarkers of brachial plexus avulsion caused neuropathic pain in male rat

**DOI:** 10.1186/s12868-022-00717-9

**Published:** 2022-05-26

**Authors:** Le Wang, Jie Lao

**Affiliations:** 1 Department of Pediatric Surgery, Affiliated Ruijin Hospital, Shanghai Jiao Tong University Medical School, Shanghai, People’s Republic of China; 2 Department of Hand Surgery, Huashan Hospital, Fudan University, Shanghai, People’s Republic of China; 3 Key Laboratory of Hand Reconstruction, Ministry of Health, Shanghai, People’s Republic of China; 4 Shanghai Key Laboratory of Peripheral Nerve and Microsurgery, Shanghai, People’s Republic of China

**Keywords:** Neuropathic pain, Brachial plexus injury, mRNA, Animal models, Potential biomarkers

## Abstract

The present study aimed to investigate the expression of mRNA in the brachial plexus avulsion neuropathic pain model and analyze biological functions. Microarray mRNA assay and reverse transcriptase quantitative polymerase chain reaction (RT-PCR) were conducted. The whole blood was collected from two groups for Microarray mRNA analysis. The predicted mRNA targets were studied by gene ontology analysis and pathway analysis. We identified 3 targeted mRNAs, including PIK3CB, HRAS, and JUN. The results showed that PIK3CB, HRAS, and JUN gene expression was increased in the control group but decreased in the neuropathic pain group. These findings indicate that certain genes may be important biomarkers for the potential targets for the prevention and treatment of brachial plexus avulsion caused neuropathic pain.

## Introduction

Nerve injury-induced neuropathic pain remains an intractable disease due to a lack of satisfactory treatment [9]. In 2017, Palma Ciaramitaro et al. [1] investigate the prevalence of neuropathic pain after traumatic brachial plexus injury. Of the 107 patients enrolled, 69% had neuropathic pain. Neuropathic pain can significantly impair function, appetite, sleep, mood, and quality of life. Brachial plexus avulsion (BPA) induces a characteristic of pain are allodynia, hyperalgesia, and persistent pain, which is often difficult to cure [10]. The pain may be manifested as burning or pressure. Pain after BPA is resistant to most pain relief treatments the exact molecular mechanisms responsible for this pathology remain unknown [3]. Previous studies demonstrated that the mRNA plays a key role in the development and maintenance of neuropathic pain [11–13].

Some authors proved that c-Jun plays a vital role in the survival of ventral horn motoneurons in adult mice [4]. HRAS gene is related to neural substructure development [23]. Previous studies have shown that PIK3CB influences the early development of neuropathy in sensory neurons [2]. The spinal cord plays an important role in the process of central sensitization [14]. Furthermore, we aimed to investigate the mRNA changes of neuropathic pain caused by the brachial plexus avulsion model, thereby providing a novel insight into the mechanism of neuropathic pain.

## Material and methods

### Animals

This study was carried out in strict accordance with the recommendations in the Guide for the Care and Use of Laboratory Animals of the National Institutes of Health. The protocol was approved by the Committee on the Ethics of Animal Experiments of the University of Fudan (GB/T 35,892-2018). All surgery was performed under sodium pentobarbital anesthesia, and all efforts were made to minimize suffering. The experiments were conducted in male Sprague- Dawley rats (n = 20, age, eight weeks; weight, 200- 250 g; supplied by the Department of Laboratory Animal Science, Fudan University, Shanghai, China).

### Surgery procedure

All surgical procedures were performed after anesthesia induced by a 1% sodium pentobarbital solution (40 mg/kg body weight). Place the rat prone on a sterilized pad with the head oriented away from the surgeon and the right forepaw abducted and extended. Use the fingertips to locate the clavicle. With a scalpel, make a 1.5 cm horizontal incision in the skin under the clavicle 2 mm. Use micro-dissecting scissors to separate the skin from the superficial fascia, exposing the pectoralis major muscle.

The pectoralis major muscle was cut paralleled with the muscle fibers to expose the brachial plexus, leaving the cephalic vein intact. The subclavian vessels were located and the upper, middle, and lower trunks were dissected. In the complete brachial plexus avulsion (BPA) group (n = 20), the upper, middle, and lower trunks were grasped with forceps and hauled out from the spinal cord. The tissue layers were then brought together, and the skin was closed with 4–0 silk sutures (Ethicon), as described previously [7].

### Animal pain tests

#### Mechanical allodynia

Mechanical allodynia was assessed by using the von Frey filaments (Stoelting, USA; bending force: 2.0, 4.0, 6.0, 8.0, 10.0, 15.0, and 26.0 g). The filaments were applied to the left forepaw. The threshold was the lowest force that evoked a withdrawal response. Each filament was applied five times. When rats showed at least two withdrawal responses to a filament, the bending force of the filament was defined as the withdrawal threshold [7].

#### Cold allodynia

Cold allodynia was assessed by an acetone spray test as described by Choi et al. [8]. 250 μl acetone was squirted onto the surface of the paw. Neuropathy rats frequently responded with a withdrawal that was exaggerated in amplitude and duration. The withdrawal responses were assessed on a scale of 3–0 points: 3 points, a vigorous response in which the rat licked the paw; 2 points, a response in which the paw has elevated the paw; 1 point, a response in which the paw had little or no weight born on it and 0 points, the paw was not moved [5].

### mRNA microarray

The whole blood was collected from the rats. Total RNA was extracted from whole blood using a QIAamp RNA blood mini kit (Qiagen) the manufacture’s instruction. RNA quality was checked with denaturing agarose gel (1.5%) electrophoresis and nucleic acid staining. Samples with 28S and 18S rRNA bands were resolved into two discrete bands that had no significant smearing below each band, and the 28S rRNA band intensity, which was approximately twice that of the 18S rRNA band, was used for subsequent procedures. Total RNA (1 µg) was labeled with Affymetrix^®^ FlashTag™, Biotin HSR RNA Labeling kits (Affymetrix, Inc., Santa clara, cA, USA). Next, samples were hybridized to a Genechip Rat Gene 1.0 (Affymetrix, Inc.) at 60 rpm, at 48 °C for 16 h. Fluorescent images of microarray slides were scanned using a Genechip^®^ Scanner 3000 7G (Affymetrix, Inc.). Microarrays were processed using an Agilent GeneArray Scanner with Affymetrix Microarray Suite version 5.0.0.032 software [5].

### Reverse transcription-quantitative polymerase chain reaction (RT-PCR) assay

RNA was reverse transcribed into cDNA using Takara PrimeScript RT master mix (RR036A; Takara Biotechnology Co., Ltd., Dalian China). RT-qPCR was performed using an ABI StepOne Plus Real-Time PCR system (Thermo Fisher Scientific, Inc.) and SYBR Premix Ex Taq II master mix (Takara Biotechnology Co., Ltd.) according to the manufacturer's protocol. The reaction system (10 µl) consisted of cDNA (1 µl), forward primers (10 µM; 0.2 µl), reverse primers (10 µM; 0.2 µl), ROX reference dye (0.2 µl), RNase-free water (3.4 µl), and SYBR-Green mixture (5 µl). The thermocycling conditions were as follows: Initial denaturation, 95 °C for 30 s, followed by 40 cycles of 95 °C for 5 s and 60 °C for 30 s. Rat actin was used as a housekeeping gene. The relative expression of genes was calculated using the 2^−∆∆Ct^ method [5].

### Bioinformatic evaluation

GO analysis was applied to analyze the function of the expression genes according to the Gene Ontology, which is the crucial function of NCBI that can organize genes into hierarchical classification and uncover the gene network based on biological process and molecular function.

Pathway analysis was applied to find out the significant pathway of the differential genes according to KEGG, Biocarta, and Reactome. Still, we turn to Fisher’s exact test and $\chi^{2}$ test to select the most significant pathway, and the threshold of significance was decided by *P*-value and FDR. The enrichment Re was calculated like the equation above [5].

### Statistical analysis

The random variance model t‑test was adopted to filter the differentially expressed mRNAs between the control and pain groups using GraphPad 5.0. Following the significance analysis and false discovery rate analysis, differentially expressed genes were selected according to their P-values. P < 0.05 was considered to indicate a statistically significant difference [5].

## Results

### mRNA microarray

Animals exhibiting significant decreases in the pain threshold (mechanical threshold decreases from 15 g pre-surgery to 8 g post-surgery and allodynia score increases from 0 pre-surgery to 2–3 post-surgery) were placed in the NP (Neuropathic Pain) group. 10 rats were doing the BPA surgery and 6 rats had neuropathic pain. There were 6 rats in the NP group. The sham-operated animals whose brachial plexus was just dissected but not used were assigned to the control group. There were 6 rats in the C group(Control group).

To functionally investigate a possible link between mRNA expression and the brachial plexus injury neuropathic pain, the differential expression of mRNA in the neuropathic pain and control group was analyzed. The whole blood was harvested from the rat after 2 weeks. The expression of 2717 mRNAs was detected between the pain and control group according to the changes: down and up. By contrast to the control group and the pain group, 1154 mRNAs exhibited decreased expression, and 1563 mRNAs exhibited increased expression. The differentially expressed mRNAs between neuropathic pain group and control group genes are shown in Table 1.

**Table 1:** The most significant upregulated genes or downregulated genes in the neuropathic pain group

Probe set ID	Gene symbol	Gene description	P-value FD
10,937,619	LOC685774	Hypothetical protein	0.0253862 − 3.68
10,937,311	Mir448	microRNA	0.0380793 − 3.55
10,936,853	Midlip1	MID1 protein	0.0390031 − 3.53
10,934,445	Ogt	GlcNAc transferase	0.0395949 − 3.4
10,929,600	Pde6d	Phosphodiesterase 6D	0.0389264 − 3.32
10,929,445	Tm4sf20	Transmembrane	0.0374896 − 3.28
10,925,373	Ube2f	Ubiquitin-conjugating enzyme E2F	0.0308392 − 3.11
10,911,048	Dapk2	Death-associated kinase 2	0.0257355 − 3.11
10,908,788	Zbtb44	Zinc finger and BTB domain containing 44	0.0406164 − 3.09
10,905,558	Rpl26	Ribosomal protein L26	0.0278983 − 2.95
10,714,907	Ifit1	Interferon-induced	< 1e−07 149.84
10,886,573	Ifi27	Interferon, alpha-inducible protein 27	< 1e−07 40.88
10,811,177	Ctrb1	Chymotrysinogen B1	< 1e−07 24.74
10,882,317	Isg15	ISG 15 Ubiquitin-like modifier	< 1e−07 12.25
10,827,820	RT1-T24-4	RT1 class I, locus T24, gene 4	< 1e−07 9.13
10,737,262	Supt4h1	Suppressor of Ty4 homolog 1	< 1e−07 6.22
10,732,592	Nprls	Nitrogen permease regulator-like 3	< 1e−07 4.7
10,785,144	Xpo07	Exportin7	< 1e−07 4.19
10,749,495	Lgals3bp	Lectin, galactoside-binding, soluble, 3 binding protein	< 1e−07 3.72
10,704,505	S1c1a5	Solute carrier family 1(neutral amino acid transporter), member 5	< 1e−07 3.69

*FD* fold change

We have found 621 GO terms with the P-value < 0.05. The top 20 GO terms, ranked by P-value, were shown in Table 2. Most of the enriched terms were about inflammatory processes involved in protein modification and regulation of biological processes. The result was similar to the GO analysis.

**Table 2:** The top 20 most significant GO terms in the neuropathic pain group

GO terms	GO name	Path-count	Enrichment trend
0,009,615	Response to virus	84	12.30162448 up
0,045,087	Innate immune response	93	10.69962082 up
0,008,150	Biological process	1408	2.582253877 up
0,043,066	Negative regulation of apoptotic	478	4.003318056 up
0,042,493	Response to drug	462	3.893443439 up
0,051,607	Defense response to virus	96	8.371938884 up
0,014,070	Response to an organic cyclic compound	230	5.158362344 up
0,008,285	Negative regulation of cell proliferation	308	4.349059161 up
0,032,355	Response to estradiol stimulus	143	6.155591427 up
0,006,954	Inflammatory response	189	5.264892783 up
0,008,150	Biological process	1408	2.44882276 down
0,006,355	Regulation of transcription DNA-depensent	681	2.8479700082 down
0,006,351	Transcription, DNA-dependent	640	2.862061601 down
0,006,886	Intracellular protein transport	167	4.838991083 down
0,015,031	Protein transport	271	3.777150973 down
0,006,412	Translation	384	3.226834158 down
0,045,944	Positive regulation of transcription from RNA polymerase II promoter	715	2.411148564 down
0,000,122	Negative regulation of transcription from RNA polymerase II promoter	499	2.699103242 down
0,015,986	ATP synthesis coupled proton transport	19	14.17739493 down
006,302	Double-strand brake repair	48	7.856639688 down

*GO* gene ontology, *Count* enriched gene numbers in each term

**Fig. 1:**
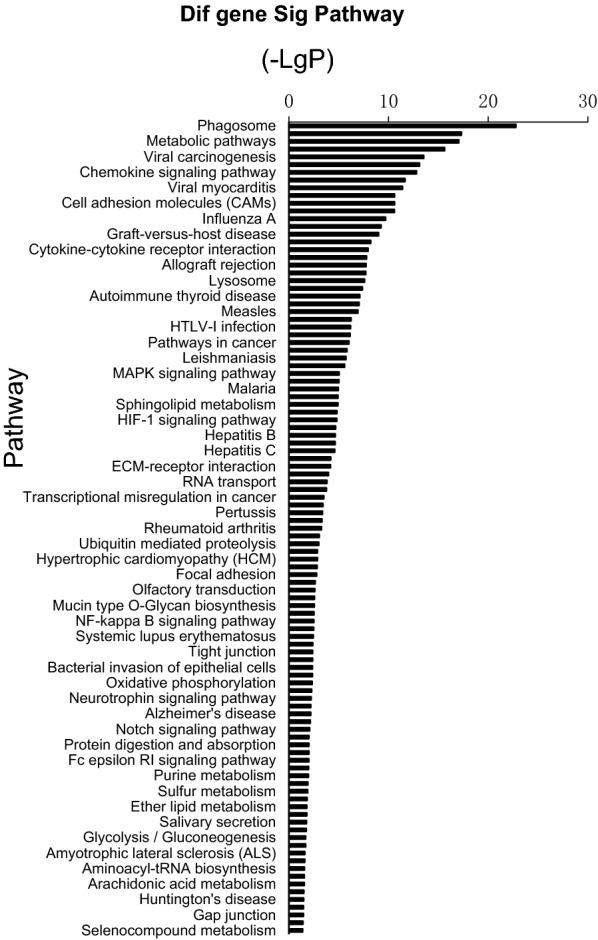
Relative expression of differentially expressed mRNA in rat whole blood in the microarray. **a** Hras were significantly down-regulated in the neuropathic pain group versus the control group after 2 weeks. **b** Jun was significantly down-regulated in the neuropathic pain group versus the control group after 2 weeks. **c** Pik3cb were significantly down-regulated in the neuropathic pain group versus the control group after 2 weeks. Data are presented as mean ± SE, *p < 0.05. NP group: neuropathic pain group. The KEGG pathway up regulation histogram. LGP (The P value is logarithmic and negative): the larger the (–LGP) value, the smaller the P value, indicating the higher the significance level of the pathway

**Fig. 2:**
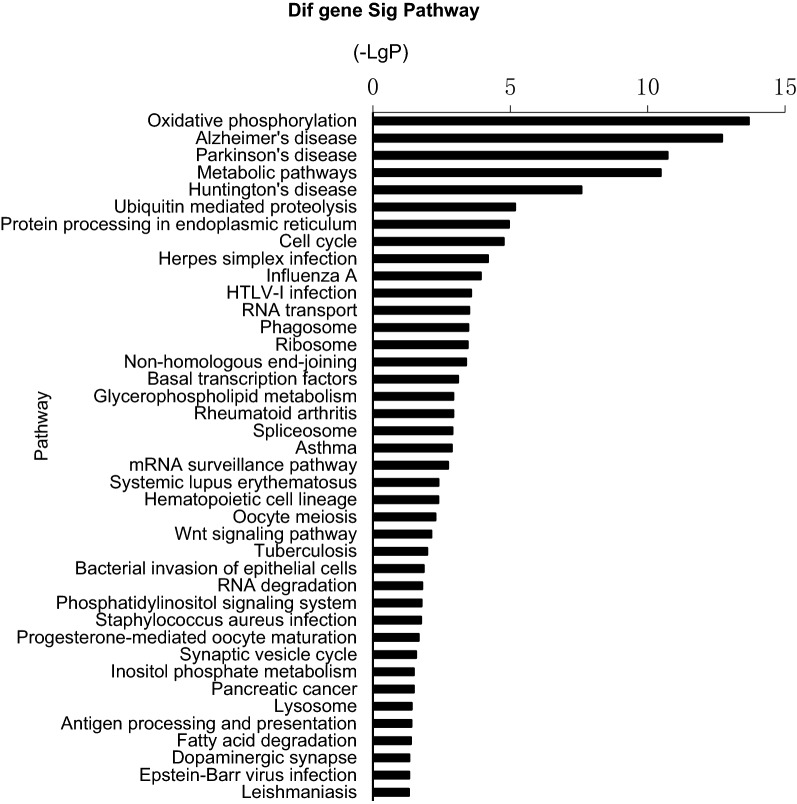
The KEGG pathway down regulation histogram. LGP (The P value is logarithmic and negative): the larger the (–LGP) value, the smaller the P value, indicating the higher the significance level of the pathway

To further investigate the functions of DEGs, we did a KEGG pathway analysis. The top 20 pathways were shown in Table 3. The KEGG pathway up regulation histogram (Fig. 1) and down regulation histogram (Fig. 2).

**Table 3:** The top 20 most significant enriched KEGG pathways

KEGG term	Pathname	Path gene count	Enrichment trend
04,145	Phagosome	196	7.810555227 up
05,168	Herpes simplex infection	218	6.320100652 up
01,100	Metabolic pathways	1272	2.768080422 up
04,612	Antigen processing and presentation	98	9.372666273 up
05,203	Viral carcinogenesis	239	5.284379834 up
05,169	Epstein-Barr virus infection	232	5.278858016 up
04,062	Chemokine signaling pathway	180	5.953378762 up
04,670	Leukocyte transendothelial migration	119	7.075444147 up
04,516	Viral myocarditis	110	7.30641939 up
04,144	Endocytosis	236	4.702880923 up
00,190	Oxidative phophorylation	162	7.981348255 down
05,010	Alzheimer’s disease	214	6.545451489 down
05,012	Parkinson’s disease	164	6.898512897 down
01,100	Metabolic pathways	1272	2.625938872 down
05,016	Huntingtin’s disease	219	4.920009198 down
04,120	Ubiquitin mediated proteolysis	136	5.149730216 down
04,141	Protein processing in the endoplasmic reticulum	165	4.571135819 down
04,110	Cell cycle	126	5.130866735 down
05,168	Herpes simplex infection	218	3.706933536 down
05,164	Influenza A	177	3.956854855 down

*KEGG* kyoto encyclopedia of genes and genomes

### PCR verification

We aimed to explore the mechanism of neuropathic pain after brachial plexus avulsion and find nerve-related mRNAs. Therefore, we mainly focused on the differentially expressed genes (DEGs) dysregulated only in the neuropathic pain groups. Three mRNAs (Pik3cb, Hras, and Jun genes) were decreased expression in the neuropathic pain group. We chose three mRNAs (Pik3cb, Hras, and Jun genes) because it involved in neural substructure development [23].

**Fig. 3:**
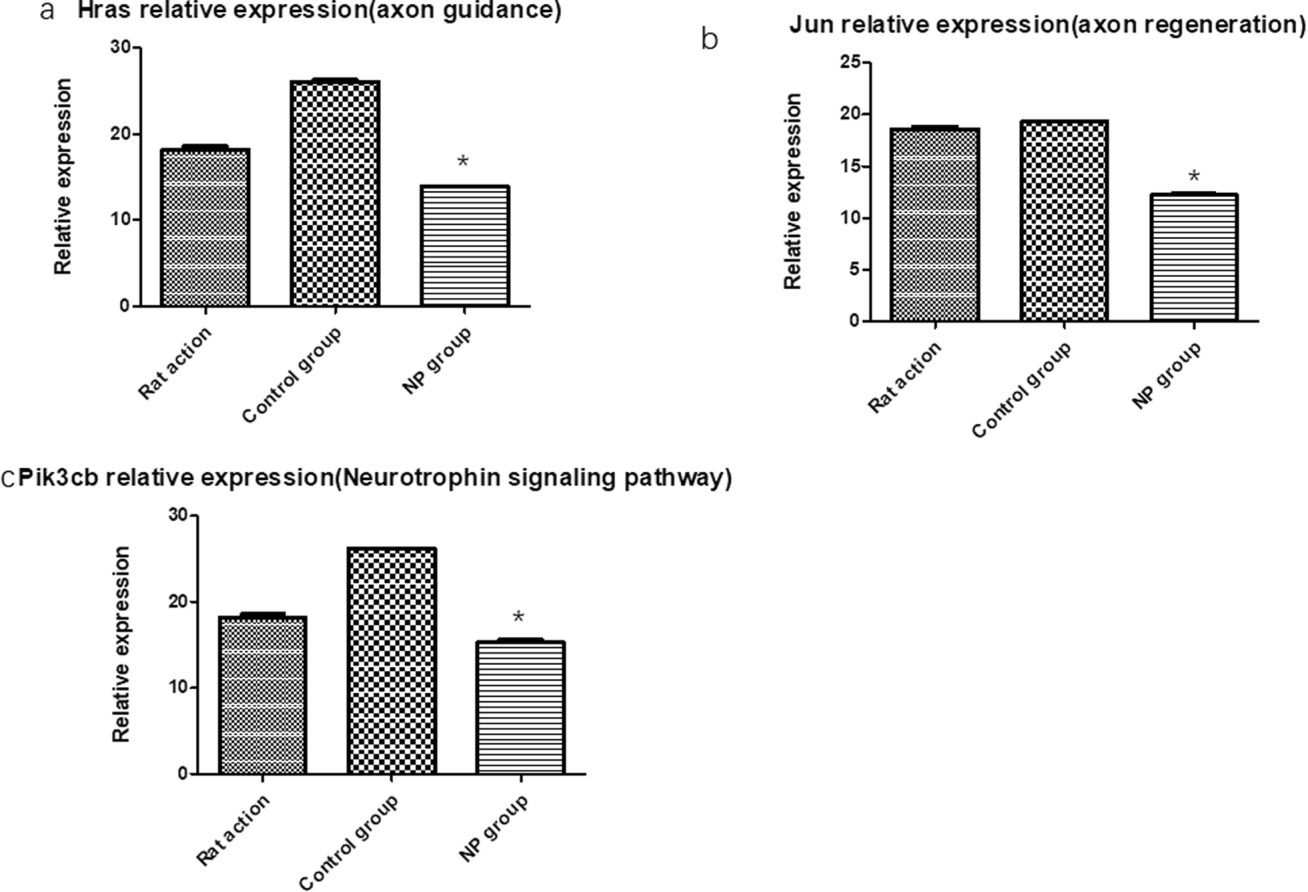
Relative expression of differentially expressed mRNA in rat whole blood in the microarray. **a** Hras were significantly down-regulated in the neuropathic pain group versus the control group after 2 weeks. **b** Jun was significantly down-regulated in the neuropathic pain group versus the control group after 2 weeks. **c** Pik3cb were significantly down-regulated in the neuropathic pain group versus the control group after 2 weeks. Data are presented as mean ± SE, *p < 0.05. NP group: neuropathic pain group

To validate the microarray results, RT-qPCR was performed for Pik3cb, Hras, and Jun genes. It was found that the relative expression of 3 mRNA among them was significantly altered, which coincided with the results of the microarray (Fig. 3).

### Bioinformatics analysis of the diff-reg mRNA

The Pik3cb, Hras, and Jun genes were intersection genes, which were involved in neuropathic pain according to GO and pathway analyses. The results showed that Pik3cb, Hras, and Jun gene expression was high in the control group but was low in the neuropathic pain group. The function of the Hras gene was synergetic in the aspect of axon guidance and the Neurotrophin signaling pathway. The Jun gene function was axon regeneration. The low expression of two genes in the neuropathic pain group was revealed that neuropathic pain is unfavorable for nerve regeneration.

## Discussion

Brachial Plexus Avulsion (BPA) has been demonstrated to be a polygenic disease and its pathogenic mechanism is associated with changes in many genes. In this study, we have used microarray to identify differentially expressed genes(DEGs) and activated signaling pathways in association with BPA-induced neuropathic pain (NP) in a rat BPA model. Experiments were only conducted in male rats because our previous study used male rats [5, 7, 11]. In the next experiment, we will compare the DEGs differences between male and female rats. Theodore Price et al. demonstrated that sex differences in the mechanisms contributing to the development and/or maintenance of pain in males and females [24]. Female-predominant DEGs in sensory neurons related to inflammatory, synaptic transmission and extra celluar matrix reorganization processes; Male-selective DEGs were linked to oxidative phosphorylation and protein/molecule metabolism and production [24]. Our results have shown similar trend.

The results of GO and KEGG pathway enrichment analyses have shown similar trends, since many enriched terms or pathways were inflammatory processes and immune responses related [27, 28]. Up to date, many evidence suggested that both axonal regeneration and functional recovery are important contributors to neuropathic pain [25, 29]. These findings are all consistent with our present study. The KEGG analysis unraveled several signaling pathways enriched in BPA(brachial plexus avulsion) model rats. Among these pathways, phagosome, chemokine signaling, and oxdative phophorylation pathways especially attracted our attention since they have been implicated in mediating chronic pain.

We showed that Jun, HRAS, and PIK3B were the nerve-related downregulated DEGs. Our results are consistent with that of previous studies [7]. Although the precise roles of the three marker genes in BPA-induced NP are not completely understood, our data highlighted the diagnostic and treatment potential of this disease.

We noticed that the most significantly enriched biological process of downregulated KEGG pathway was Alzheimer’s disease, Parkinson’s disease and Huntington’s disease, etc. At this stage, we have no idea how these processes might be related with neuropathic pain. How these biological processes might be related with neuropathic pain is unknown and still needs further investigation.

It will be very interesting to further this study into BPA patients. Microarray technology can be reliable and useful for identifying novel targets for clinical diagnostic and therapeutic approaches. This technology can be used in pancreatic cancer and renal clear cell carcinoma for diagnosis and effective therapy [15, 17].

We found that three genes expressed decreased and were related to nerve regeneration. Some authors proved that the downregulation of c-Jun gene expression is not conducive to the survival of motoneurons. HRAS might serve specific roles in the development and maintenance of nervous tissues [6]. In our study, the Metabolic signaling pathway and Phagosome signaling pathway are involved in BPA, which play a very important role in BPA-induced NP. In the peripheral nervous system, recent studies suggested that the nerve-related gene plays an important role in neuropathic pain after spinal cord injury [16]. Some authors suggested that there is a high possibility of neuropathic pain caused by nerve damage [18, 19]. The transcriptome changes play an important role in neuropathic pain [13]. So our research is meaningful and feasible. Ji-An Yang et al. [20] proved that Jun is a potential indicator for neuropathic pain. Despite increasing knowledge and ongoing study, the precise molecular mechanisms of neuropathic pain caused by brachial plexus injury remain largely unknown. Numerous studies show a significant modification of gene expression as a consequence of nerve injury. A study by Timo et al. reported that miRNAs-494, -720,-690, and -668 showed the highest signal intensities in the rat spinal cord [21]. The exosomes with Ccl3 can be efficiently detected in peripheral blood. Guan Zhang et al. [22] proved that Ccl3 can be used as a potential prognostic target for the diagnosis and treatment of spinal cord injury-induced chronic neuropathic pain in clinical applications. The microarray analysis of DEGs and pathway indifferent section by GO and KEGG suggests another method and strategy to research the target gene and pathway of nerve-related disease [22]. William Renthal et al. [25] proved that nerve injury induces distinct transcriptional responses in non-neuronal DRG cell types. The important roles of mitochondrial proteins and reactive oxygen species (ROS) metabolism, especially oxidative phosphorylation, in nerve and tissue degeneration/regeneration is highlighted by a robust literature of hundreds of papers [30]. Enhancing the mitochondrial oxidative phosphorylation process can be an effective approach for recovery from nerve damage and degeneration.

In summary, our studies indicated that Jun, HRAS, and PIK3B might serve a significant role in neuropathic pain and nerve regeneration. The three genes were downregulated in the spinal cord in NP rats after brachial plexus avulsion. Furthermore, KEGG analysis found that Metabolic pathways with significance were identified. Microarray data can provide a great source of information in pain research [26]. These findings indicate that certain genes may be important biomarkers for the potential targets for the prevention and treatment of brachial plexus avulsion caused neuropathic pain. Several limitations should be acknowledged in our study. First, the sample size was relatively small. Besides, the results were all based on a rat model. In the future, we will perform some more in-depth studies around nerve-related genes.

## Data Availability

All data generated or analyzed during this study are included in this published article.
